# Regenerative effect of mesenteric fat stem cells on ccl4-induced liver cirrhosis, an experimental study

**DOI:** 10.1016/j.amsu.2020.10.045

**Published:** 2020-10-24

**Authors:** Fatemeh Dehghani Nazhvani, Iman Haghani, Seifollah Dehghani Nazhvani, Fatemeh Namazi, Abbas Ghaderi

**Affiliations:** a Bone and Joint Diseases Research Center, Shiraz University of Medical Science, Shiraz, Iran; b Department of Veterinary Surgery, School of Veterinary Medicine, Shiraz University, Shiraz, Iran; c Department of Pathology, School of Veterinary Medicine, Shiraz University, Shiraz, Iran; d Shiraz Institute for Cancer Research, School of Medicine, Shiraz University of Medical Sciences, Shiraz, Iran

**Keywords:** Hepatic cirrhosis, Liver fibrosis, Animal model, Adipose-derived mesenchymal stem cells, ADSCs, Multipotent adipose-derived stem cells, MSCs, Mesenchymal stem cells, CCL4, Carbon tetracholoride, IP, Intraperitoneal, AST, Aspartate transaminase, ALT, Alanine transaminase, PBS, Phosphate buffered saline, EDTA, Ethylenediaminetetraacetic acid, α-MEM, Minimum essential medium α, FBS, Fetal bovine serum, IM, Intramuscular, CNS, Central nervous system, HGF, Hepatocyte growth factor, VEGF, Vascular endothelial growth factor

## Abstract

**Background:**

Liver cirrhosis is a chronic disease in which normal liver tissue is replaced by fibrous tissue, leads to liver malfunction. Although transplantation is the most certain cure, stem cell therapies are shedding light on efforts to regenerate cirrhotic liver. The purpose of this study was to evaluate the regenerative potential of mesenteric fat stem cells in CCL4-induced liver cirrhosis in an animal model.

**Methods:**

Thirty rats were treated with the mixture of CCL4 and olive oil intraperitoneally by a dose of 0.2 ml (0.1 ml CCL4 and 0.1 ml olive oil) every other day for 16 weeks till cirrhosis signs appeared. Fifteen rats were randomly selected as control group. Others treated by mesenteric fat derived mesenchymal stem cells transferred into the liver parenchyma.

**Results:**

After 5 weeks, rats received stem cells had improved clinically by increased movements, appetite, improvement in overall behavior and decreased abdomen size. Histopathologically, liver cells showed state of regeneration and forming new colonies.

**Conclusion:**

Liver cirrhosis was induced. The mesenchymal stem cells derived from mesenteric adipose tissue could improve hepatic status of the rats, as cirrhotic livers were regenerated back into normal appearing parenchyma. Rats’ clinical behavior also reached healthy status.

## Highlights

• The study evaluated the regenerative potential of mesenteric fat stem cells in cirrhotic livers.
• All aspects of a patient (in this study animals) including clinical behavior, paraclinical investigation, serum parameters and histopathologic views were analysed before and after the surgical treatment.
• Both intravenous and intraparenchymal administration of stem cells was the new method used in this study.

## 1 Introduction

Liver cirrhosis is a chronic disease in which normal liver tissue is replaced by fibrous tissue, leads to liver malfunction. Cirrhosis often occurs due to many factors such as alcohol abuse, hepatitis (type B and C), fatty liver, drugs, toxins, several congenital diseases (fibrocystic liver disease, genetic defects, Wilson's disease and hemochromatosis), long-term obstruction of bile ducts for any reason and more [1,2]. Medications like prednisolone and azathioprine are used to treat autoimmune inflammation of the liver; also interferons and antiviral agents are used to treat hepatitis B and C, ursodeoxycholic acid for treatment of primary biliary cirrhosis, trientine and penicillamine for treatment of Wilson's disease [2].

As the disease progresses, treatments focused toward to the management of symptoms and complications. These complications include ascites, visceral hemorrhage, hepatic encephalopathy and the most important of them, cirrhosis [1,2]. Although transplantation is the most certain cure (FDA-approved) in the case of cirrhosis, it has its own complications and difficulties such as inadequate donors.

Stem cell therapies are shedding light on efforts to regenerate cirrhotic liver. Multipotent adipose-derived stem cells (ADSCs) have received significant attention as regenerative medicine for liver fibrosis owing to their advantages over stem cells with other origins [3]. They play this role by two major potential mechanisms; one is the improvement of the microenvironments through paracrine effects, and the other is the replacement of functional hepatocytes [4].

Then mesenchymal stem cells (MSCs) can suppress inflammatory responses, decrease hepatocyte apoptosis, increase hepatocyte regeneration, regress liver fibrosis and enhance liver functionality. Despite these benefits, an issue remain; MSCs also have the capacity to promote tumor cell growth which should be taken into consideration [5]. The purpose of this study was to evaluate the regenerative potential of mesenteric fat stem cells on carbon tetracholoride (CCL4) induced liver cirrhosis in an animal model.

## 2 Material and methods

Thirty three adult male Sprague Dawley rats were used in this study. The study was approved by the Research Ethics Committee of Shiraz University (# 94–1394) and its research protocol was checked and registered by the university ministry of research. The work has been reported in accordance with the ARRIVE guidelines (Animals in Research: Reporting In Vivo Experiments) [6]. The rats were housed in standard cages in 22 °C, 12 h light and 12 h darkness with proper food and water. Then they were treated with the mixture of CCL4 (Merck, Germany) and olive oil intraperitoneally (IP) by a dose of 0.2 ml (0.1 ml CCL4 and 0.1 ml olive oil) every other day for 16 weeks till cirrhosis signs appeared. Eighteen rats were selected as control group by simple randomization technique. Others, fifteen rats, treated by mesenteric fat derived mesenchymal stem cells. To confirm cirrhosis, sonographic evaluation and serum biochemical analysis were done on all rats and three rats were picked up randomly from control group, euthanized for gross necropsy and histopathologic evaluation of the liver cirrhosis.

### 2.1 Sonographic evaluation

All rats sedated and shaved to locate their liver using convex probe (size 3.5) on the abdomen skin. The liver surface, volume and echogenic quality were evaluated before CCL4 injection, 16 weeks later and five weeks after injection of stem cells.

### 2.2 Serum analysis

Blood samples were taken directly from the heart of all rats before CCL4 injection, 16 weeks later and five weeks after injection of stem cells to test their aspartate transaminase (AST), alanine transaminase (ALT), alkaline phosphatase, albumin, bilirubin and total protein.

### 2.3 Preparation of stem cells

A rat was anesthetized, restrained on its dorsal recumbence, under aseptic condition the linea alba was incised and all the mesenteric fat was separated and transferred to the laboratory under standard conditions. The tissues were smashed by mechanical tools, centrifuged, and the pellets transferred to phosphate buffered saline-ethylenediaminetetraacetic acid (PBS-EDTA) solution with 1% penicillin/streptomycin and 1% fungizone (both from Gibco/Invitrogen Company, Carlsbad, California, USA). The tissues were broken under sterile conditions by using enzymatic digestion while occasionally shaking for 1 h in a solution of 3 mg/mL collagenase type 1, and 4 mg/mL dispase type 2 (both from Sigma, St. Louis, Missouri, USA). The resulting single-cell suspension was passed through a 70-μm cell filter (Biosciences BD, San Jose, California, USA) and centrifuged for 10 min to remove the supernatant remaining enzymes.

The single-cell suspensions were seeded onto cultivation flasks within the minimum essential medium **α** (α-MEM) along with 4 mM GlutaMax, 100 U/ml penicillin, 100 mcg/ml streptomycin and 20% fetal bovine serum (FBS) (all from Gibco/Invitrogen). The cells were cultured for 72 h at 37 °C in 5% CO2 and 90% humidity. Then, the free cells and residuals were removed and fresh medium was added to the adherent cells. The medium was changed twice a week until the flask reached 80% confluence. The cells were then released with Trypsin- EDTA (Gibco/Invitrogen) and the second culture was performed. They were passaged three times before they could be used for injection.

### 2.4 Stem cells administration

After cirrhosis was confirmed, The other fifteen Rats were anesthetized by intramuscular (IM) injection of combination of 5–10 mg/kg xylazin (Xyla, Interchemie, Holland) and 80–100 mg/kg ketamine (Alfasan, Woerden Holland). The midline abdomen was prepared for aseptic surgery. The linea alba was incised and the portal vein was located, then the stem cells (1 ml containg 10^6^ cells) were injected into the portal vein. Few drops of Hemoliq (Hemoliq, Technew- Brasilia) (containing a mixture of aluminum chloride and hydroxyquinoline and propylene glycol) was dropped on the location of needle insertion to prevent hemorrhage, then needle was removed. In addition, stem cells were also injected in few sites of liver parenchyma of all fifteen rats. Then the abdominal wall was sutured routinely. Five weeks after injection of stem cells, the rats were euthanized and samples of liver tissue were collected in 10% buffered formaldehyde containers for histopathologic investigation. The samples were stained by H&E and Masson Trichrome [7] and studied in a single blind manner by the pathologist.

### 2.5 Histopathologic evaluation

The degree of necrosis was scored as: (0) no necrosis; (1) small groups of hepatocytes showing some degree of necrosis; (2) complete centero-lobular necrosis in less than 25% of liver lobs; (3) complete centero-lobular necrosis in more than 25% and less than 50% of liver lobs; (4) complete necrosis in more than 50% of the liver lobs.

Also, the degree of fibrosis was scored as: (0) no fibrosis; (1) collection of collagen fibrils around the central liver vein but no liver cell fibrosis; (2) fibrosis extended from the central vein toward the liver lobs but not crossed the lobs yet; (3) strands of fibrosis extended between the central vein and portal vein has joined together; (4) cirrhosis along with the hepatocytic nodules surrounded by fibrosis.

All clinical results reported as descriptive data. Quantitative results were analysed by one-sample repeated measures ANOVA and Sidak post hoc test by SPSS software version 24. P < 0.05 were considered statistically significant.

## 3 Results

**Fig. 1:**
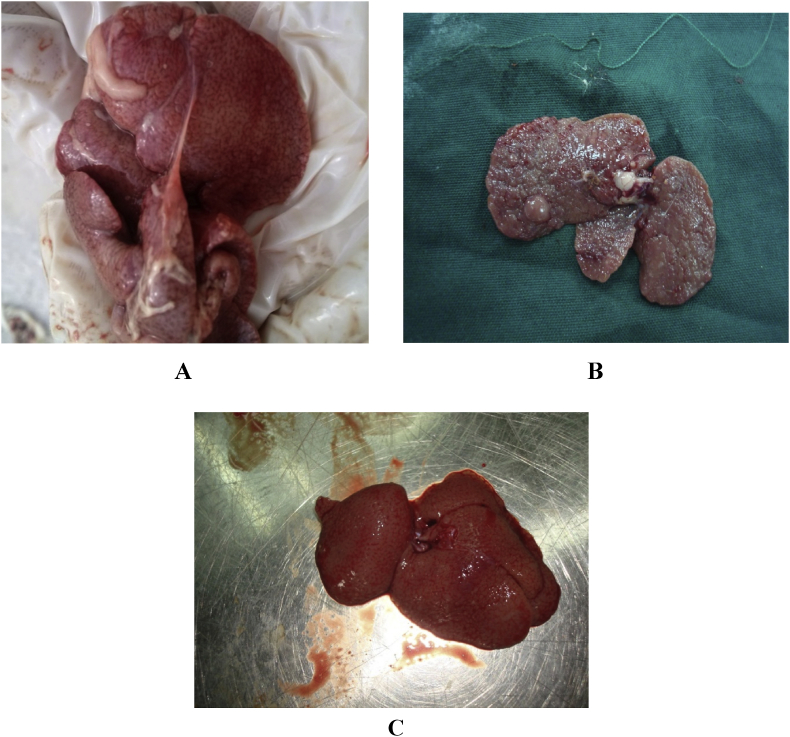
Cirrhotic liver after 16 weeks of CCL4 injection. Note the pale fatty liver, adhesions, rounded hepatic margins and micro nodular surface (A), liver Cirrhosis not treated. Note the advanced macro nodular surface of the liver (B), and liver cirrhosis treated by stem cells (5 weeks post injection), note the smooth shiny red surface without nodules (C).

**Fig. 2:**
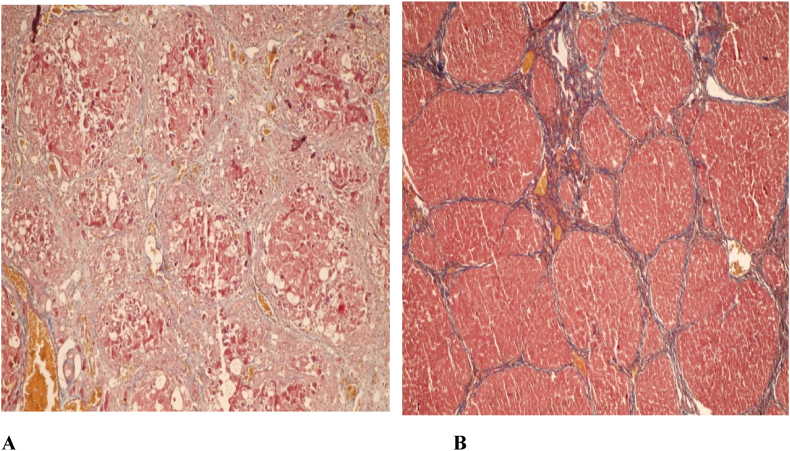
Histopathologic view of liver cirrhosis in rat, note the fibrosis and fat cells in the hepatic macro nodules and necrotic paranchyme (100X, Masson's Trichrome stain) **(A)**; Cirrhotic liver treated by stem cells injected into the hepatic parenchyma and portal vein. Note no fibrosis, no fat cells and limited micronodules (100X, Masson's Trichrome stain) (**B)**.

All the rats received CCL4 and olive oil exhibited fatigue, depression, lethargy, lack of exercise, unsociable mode, poor appetite, abdominal distension and skin bruising which was related to liver cirrhosis. Sonographic evaluation of cirrhotic livers showed rugged inequal signals of liver surface plus high echogenicity, heterogeneity in liver paranchyme, free fluid in peritoneum and hyperechoic lines of fibrotic areas; Confirmed by gross pathological examination which revealed nodular, pale yellowish appearance of the liver (due to fatty change), rounded liver margins and leathery consistency of their surface due to increased fibrosis (Fig. 1A and B & 1C). Microscopic examinations also revealed diffuse extensive fibrosis (100% of cases with score 3 and 4 of fibrosis), areas of fatty change and necrotic disorganized hepatic parenchyme (100% of cases with score 3 and 4 of necrosis) (Fig. 2A).

Serum biochemical analysis showed increase in all measurements except albumin, 16 weeks after CCL4 injection. The difference in total bilirubin was significant. These amounts were dropped toward baseline except AST in the treated group after 5 weeks of stem cell injection (Table 1).

**Table 1:** Mean amount of serum biochemical elements in rats before CCL4 injection, after 16 weeks of cirrhosis induction and 5 weeks after stem cell administration.

	Bilirubin Total mg/dl	Bilirubin Direct mg/dl	Albumin mg/dl	Total Protein mg/dl	AST^b^ IU/ml	ALT^c^ IU/ml	Alkaline Phosphatase IU/ml
**Before CCL4 injection**	0.15 ± 0.01	0.06 ± 0.00	3.36 ± 0.2	6.99 ± 0.85	213.0 ± 23.0	94.0 ± 4.0	766.5 ± 35.2
**16 wks after injection**	0.39 ± 0.1^a^	0.08 ± 0.01	3.20 ± 0.0	7.1 ± 0.1	234 ± 22.0	109 ± 6.0	779.83 ± 15
**5 wks after stem cell injection**	0.16 ± 0.06^a^	0.05 ± 0.00	4.0 ± 1.1	8.56 ± 1.6	279.75 ± 26	90.5 ± 4.0	448.0 ± 107

a Significant difference between groups (p < 0.05).

b Aspartate transaminase.

c Alanine transaminase.

Clinical results of cirrhotic rats treated by the mesenchymal stem cells included increased movements and appetite, decreased abdomen size and also improvement in overall behavior. Sonographic data illustrated decrease in liver surface inequality and parenchymal echogenicity beside lower amounts of hyperechoic lines and peritoneal free fluids. Histopathologic results of treated rats indicated that although there exists some degree of liver fibrosis in many cases, however the structural parenchymal lesions were not present showing state of regenerating hepatic cells and forming new colonies after injection of stem cells (Fig. 2B). Sections of cirrhotic group not receiving the treatment showed severe fibrosis (100% of cases with score 3 and 4), fatty change and multi acinar necrosis (100% of cases with score 3 and 4) which was compatible with clinical presentation of liver failure. Sections of treated group showed mild fibrosis (most with score 1 and few with score 2), no evidence of any parenchymal damage, no necrosis (all with score 0), no fatty change and no inflammation.

## 4 Discussion

Liver functions comprise over 500 procedures to protect the body system of the living creatures, including metabolism of the absorbed materials from the intestines, storage of the processed materials in the cells, production of many proteins, detoxification of unnecessary food materials, drugs and alcohols and many more. Therefore if the liver is damaged by cirrhosis we will observe weakness, anorexia, skin rash, discolorations, edema and in advanced cases there will be infectious peritonitis, esophageal hemorrhage, ascites and nervousness due to undigested ammonia and its impact on central nervous system (CNS) [8].

We could induce liver cirrhosis by the ligating of the bile duct as Marques et al. did; they found that ligation of the bile duct induces liver cirrhosis in shorter time than CCL4 administration (9 weeks vs. 16 weeks) [9]. But this was not considered in this study, since the method would develop many structural changes around the ligated bile duct and liver; so it would have not been possible to see the effects of stem cell or any other drugs well. Addition of olive oil to CCL4 lets gradual absorption of CCL4 from the peritoneal cavity.

There have been many researches trying to reverse the liver cirrhosis in animal models such as studies on the preventive effect of curcumin on liver cirrhosis induction in rat models by CCL4, which revealed that daily oral administration of curcumin improves liver cirrhosis and fibrosis [10,11]. In another study, the positive preventive effect of the coffee on rats' cirrhotic liver induced by CCL4 was evaluated [12]. Uzma et al. induced liver cirrhosis in rats by CCL4 and treated them by red wine demonstrated that red wine has powerful protective impact on cirrhotic liver due to its antioxidant effect, activation of immune system and anti-inflammatory effects [13].

Administration of stem cells for treatment of liver failure and cirrhosis were also assessed in many studies [14]. Takami et al. used bone marrow derived stem cells to treat chronic liver diseases and showed improvement in clinical outcomes [15]. Stem cells derived from amniotic membrane and bone marrow could treat liver fibrosis in rats, which suggests the potential effects of these stem cells for liver regeneration and reconstruction [16]. Also, autologous bone marrow derived stem cells could treat liver cirrhosis as a safe procedure [17,18]. And the health-related quality of life was studied in patients who received stem cell therapy for end-stage liver disease [19].

In the present study, we used mesenchymal stem cells derived from the mesenteric fat to treat the liver cirrhosis induced by CCL4 in a period of 16 weeks. These stem cells were able to prevent cirrhosis advancement, they could transform the fat colonies to hepatocyte colonies, reduce the connective tissue in the parenchyma and transform them to hepatic cells, so that the liver color changed from creamy yellow to nice liver red color. The liver surface nodular appearance was also changed to normal smooth surface. The stem cells were able to alter the clinical signs of liver cirrhosis so that the rats looked healthier, more alert, more sociable, showing more appetite. These clinical signs appeared after 30 days post stem cell injection.

It is suggested to extend post-op evaluation to 60 days in future studies to have better clinical and pathological assessments. We also injected the stem cells only once, and it is not clear that any second injections would be beneficial to cirrhotic conditions or may compromise the state of previous injected stem cells and their newly laid down tissues.

All previous studies have been used stem cells derived from bone marrow [15,20], umbilical cord [21] the liver itself [22] or hepatic cell line [23] and the amniotic membrane [16], but in this study we used mesenteric adipose tissue to harvest stem cells and applied a modified technique which proved good results clinically and histopathologically. Few animal studies administered adipose stem cells (ADSC) for hepatic regeneration with different methods [24,25]. Sakai et al. also enrolled a clinical trial in 2017 based on intrahepatic arterial infusion of autologous subcutaneous adipose tissue stem cells for liver cirrhosis, but it is not completed yet [26]. It seems that the therapeutic effect on the symptoms of cirrhosis is based on ADSC-secreted growth factors such as hepatocyte growth factor (HGF) & vascular endothelial growth factor (VEGF), anti-inflammatory effects on hepatic stellate cells, and the anti-fibrotic and angiogenic effects of ADSC-secreted proteins [27].

In most researches they injected the stem cells into portal vein, but this study tried to inject stem cells directly into cirrhotic liver parenchyma beside portal vein injection, because of high portal vein pressure due to cirrhosis. This technique have some advantages such as avoiding much manipulation of high pressure portal vein and its subsequent hemorrhage, preventing cardiac arrhythmias and finally patient fatality. Also it will give us opportunity to inject the prepared stem cells directly into the liver parenchyma from the abdominal wall without opening the peritoneal cavity using ultrasound or CT scan guide, which should be investigated in future.

These studies are very attractive for developing effective anti-cirrhotic therapies. Researches are needed to determine the most valuable cell source, culture condition, cell number, administration frequency, and administration route, at low cost for treating liver diseases [28]; Also to generalize the results to other species or experimental conditions.

## 5 Conclusion

Liver cirrhosis was induced by IP injection of CCL4 for 16 weeks in rats. The mesenchymal stem cells derived from mesenteric adipose tissue could improve hepatic status of the rats as their cirrhotic livers were regenerated back into normal appearing parenchyma by injection of stem cells into the portal vein and hepatic parenchyma.

## Conflicts of interest

Authors declare no conflicts of interests.

## Author's contributions

All authors participated in all parts of this research and all read and approved the final manuscript.
